# Inferring clonal structure in HTLV-1-infected individuals: towards bridging the gap between analysis and visualization

**DOI:** 10.1186/s40246-017-0112-8

**Published:** 2017-07-11

**Authors:** Amir Farmanbar, Sanaz Firouzi, Wojciech Makałowski, Masako Iwanaga, Kaoru Uchimaru, Atae Utsunomiya, Toshiki Watanabe, Kenta Nakai

**Affiliations:** 1 Department of Computational Biology and Medical Sciences, Graduate School of Frontier Sciences, The University of Tokyo, Kashiwanoha, Kashiwa-shi, Chiba Japan; 2 Laboratory of Functional Analysis in silico, Human Genome Center, The Institute of Medical Science, The University of Tokyo, Tokyo, Japan; 3 Institute of Bioinformatics, Faculty of Medicine, University of Muenster, Muenster, Germany; 4 Department of Frontier Life Science, Graduate School of Biomedical Sciences, Nagasaki University, Nagasaki, Japan; 5 Department of Hematology, Imamura General Hospital, Kagoshima, Japan; 6 Department of Advanced Medical Innovation, St. Marianna University Graduate School of Medicine, Kanagawa, Japan

**Keywords:** Data-driven modeling, Adult T cell leukemia, Human T cell leukemia virus type 1, Integration site, Clonal expansion, High-throughput sequencing, Prognostic indicator

## Abstract

**Background:**

Human T cell leukemia virus type 1 (HTLV-1) causes adult T cell leukemia (ATL) in a proportion of infected individuals after a long latency period. Development of ATL is a multistep clonal process that can be investigated by monitoring the clonal expansion of HTLV-1-infected cells by isolation of provirus integration sites. The clonal composition (size, number, and combinations of clones) during the latency period in a given infected individual has not been clearly elucidated.

**Methods:**

We used high-throughput sequencing technology coupled with a tag system for isolating integration sites and measuring clone sizes from 60 clinical samples. We assessed the role of clonality and clone size dynamics in ATL onset by modeling data from high-throughput monitoring of HTLV-1 integration sites using single- and multiple-time-point samples.

**Results:**

From four size categories analyzed, we found that big clones (B; 513–2048 infected cells) and very big clones (VB; >2048 infected cells) had prognostic value. No sample harbored two or more VB clones or three or more B clones. We examined the role of clone size, clone combination, and the number of integration sites in the prognosis of infected individuals. We found a moderate reverse correlation between the total number of clones and the size of the largest clone. We devised a data-driven model that allows intuitive representation of clonal composition.

**Conclusions:**

This integration site-based clonality tree model represents the complexity of clonality and provides a global view of clonality data that facilitates the analysis, interpretation, understanding, and visualization of the behavior of clones on inter- and intra-individual scales. It is fully data-driven, intuitively depicts the clonality patterns of HTLV-1-infected individuals and can assist in early risk assessment of ATL onset by reflecting the prognosis of infected individuals. This model should assist in assimilating information on clonal composition and understanding clonal expansion in HTLV-1-infected individuals.

**Electronic supplementary material:**

The online version of this article (doi:10.1186/s40246-017-0112-8) contains supplementary material, which is available to authorized users.

## Introduction

Clonal expansion in neoplasms is accepted as a general feature of a broad range of tumors and has been addressed from different perspectives [1–5]. Detecting clones, especially in the tumor’s early appearance, and determining the factors that affect those clones should soon have a substantial role in early diagnosis and early therapeutic intervention [3]. Monitoring the clonal expansion of most malignancies is feasible based on the analysis of genetic abnormalities within progressing and/or already developed stages; however, tracking early events has proven challenging [6]. Similar to other types of cancer, clonal expansion of abnormal cells is a hallmark of adult T cell leukemia-lymphoma (ATL), an aggressive T cell malignancy [7]. ATL is a unique neoplasm that is directly caused by infection with human T cell leukemia virus type 1 (HTLV-1) and manifests after a long latency period [8–11]. The course of disease can be monitored from early asymptomatic stages of infection to the final stages of fully developed malignancy [12]. The HTLV-1 integration site, i.e., the position at which the provirus inserted into the host genome, defines individual virus-infected cells and can be used as a marker to characterize ATL clones, as well as to monitor the clonal composition of infected individuals [13–17]. Each host cell has a unique and single integration site [18], and a population of cells with the same integration site originated from the same ancestor cell and is considered a clone [14, 15]. The clonal composition of HTLV-1-infected individuals has been investigated by a broad range of methods, including Southern blotting [7, 13], inverse PCR [16, 19–21] and linker-mediated PCR [22, 23]. Recently, by taking advantage of next-generation sequencing (NGS) technology, researchers have devised two main methods, shear sites [14] and tag systems [15], for high-throughput analysis of clonality based on provirus integration sites. These methods enable an improved understanding of genome-wide integration sites and open up new avenues to quantify clone sizes [14, 15, 20, 24–27]. In this study, we used the data derived from the tag system, which measures clone sizes without using statistical estimations [15].

In the clinical setting, the vast amounts of data rapidly generated by NGS technologies require efficient and accurate processing, analysis, management, interpretation, visualization, and conversion to useful knowledge for rendering more accurate patient outcomes [28]. In response to this demand, interdisciplinary approaches, such as the development of mathematical models in medical research, have been successfully applied over recent years. Mathematical and computational models have invaluable potential to increase the understanding of tumor development and hold promise to suggest new ways to improve the efficacy of therapeutic interventions [4, 29, 30]. To extract reliable fundamental meaning from a mathematical model and strengthen its predictive power, real experimental data must be introduced [31, 32]. Unlike other types of malignancies for which broad ranges of mathematical models are available [33], to our knowledge, there are few mathematical models for ATL [34–41], and only one visual model illustrating clonal expansion [27]. However, access to NGS data and the ability to retrieve clone size and integration site information have provided new opportunities to model clonal expansion in ATL. We recently quantified ATL clones, defined clonality patterns and categorized observed clones using threshold criteria based on clone size [27]. The conventionally described polyclonal, oligoclonal and monoclonal patterns [13, 19] can be intuitively reflected by combinations of very small, small, big, and very big (VS, S, B, and VB) clone sizes using these categorizing criteria [27]. Moreover, we showed that clone size can be a risk indicator for development and progression of ATL. Among asymptomatic carriers (ACs), individuals with polyclonal patterns remain as ACs over time, whereas those with atypical patterns of monoclonal or oligoclonal (VB or B) clones exhibited disease progression [42].

**Fig. 1:**
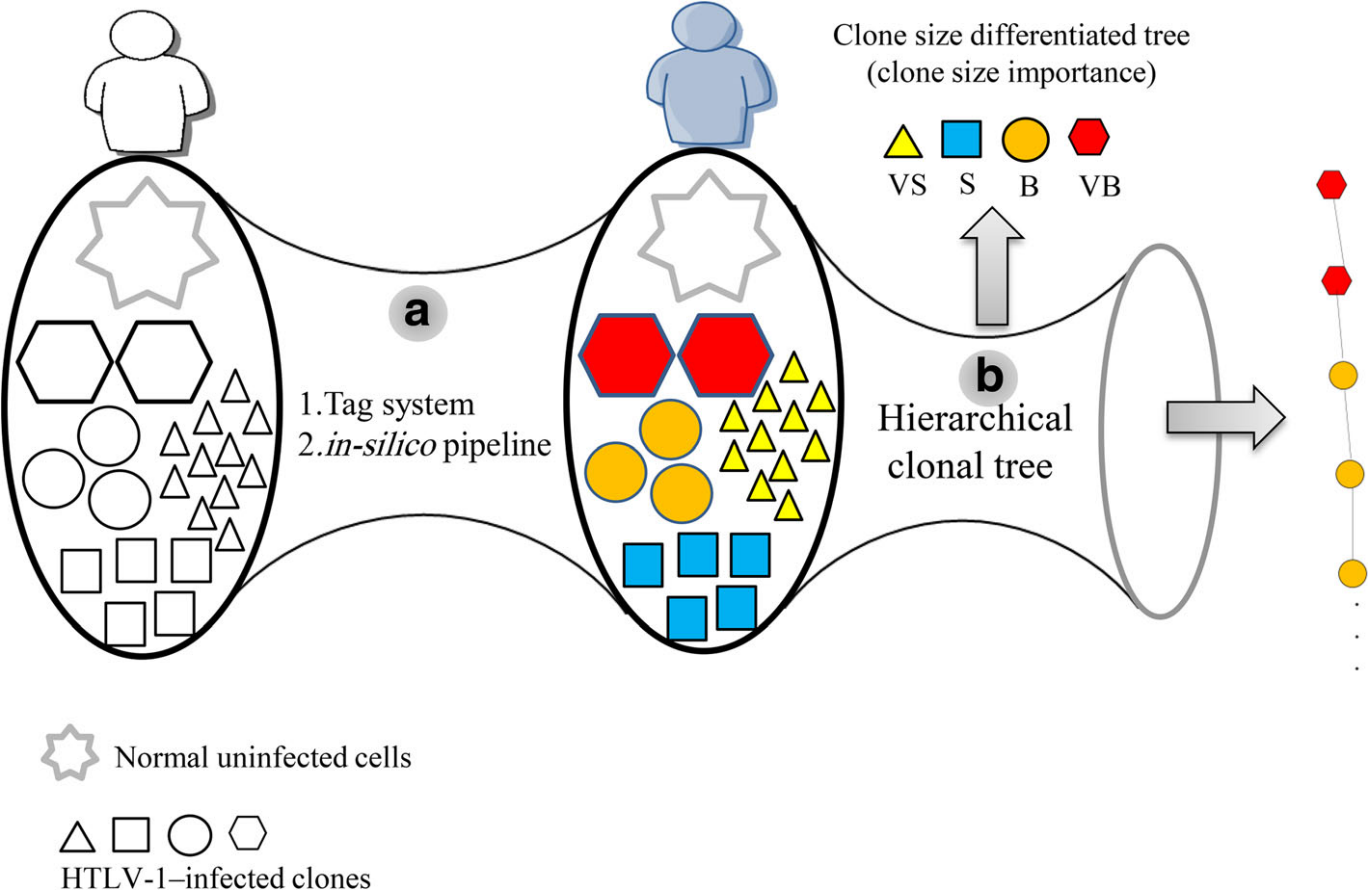
Overview of clonality analysis and visualization of results. The workflow consists of two main bottlenecks. In bottleneck **a**, our original tag system was used to isolate HTLV-1 integration sites in infected individuals and measure the size of clones. Subsequently, our in silico pipeline was used to analyze the high-throughput sequencing data. In bottleneck **b**, which is the main focus of this manuscript, the observed clone sizes were classified and visualized using different shapes and colors using the concept of hierarchical tree structure

Tree theory belongs to discrete mathematics [43, 44] and can handle the simple representation of biological data and can be used for effective data organization. Tree theory provides informative and graphical representation of concepts to readily illustrate similarities and relationships and to improve the comparison of results across samples [45]. In the present study, we developed a hierarchical tree model using both cross-sectional and longitudinal data of HTLV-1 clonal expansion. Our main goals were to display the relationship between the size and combination of clones and to visualize biological information hidden in clonality data derived from HTLV-1-infected individuals. Figure 1 illustrates a general overview of our study for representing the clonal status of each patient via hierarchical tree structures. The development of a simple model based on observed clonality data should lead to a deeper understanding of ATL clonal expansion and should enable prediction of the prognosis of infected individuals.

## Methods

In total, 60 clinical samples from 29 individuals were used, including 9 cross-sectional samples [27], 4 longitudinal samples, and 47 longitudinal samples described in [20] and [42], respectively. All clinical materials were collected by the Joint Study on Predisposing Factors of ATL Development (JSPFAD) [46, 47]. HTLV-1-infected individuals were diagnosed based on the Shimoyama criteria [48]. All sample information is provided in Additional file 1: Table S1.

Across all samples, a tag system was used to isolate integration sites and measure clone sizes. All details for experimental design and protocols are provided in [15]. In brief, BLAST searches against the HTLV-1 long terminal repeat were used to detect viral integration sites [49]. Human DNA regions flanking the integration sites were identified by alignment against hg19 using Bowtie [50]. The quality of sequencing outputs was checked with the FastQC tool [51]. Information regarding sequencing outputs and read quality is provided in Additional file 2: Figure S1. Two million mapped reads were used to measure clone sizes. We categorized observed clone sizes into four distinct groups: very small (VS, 1–128 cells), small (S, 129–512 cells), big (B, 513–2048 cells), and very big (VB, >2048 cells) [27]. We used the ordered m-ary tree to visualize the observed data. For a detailed explanation, see Additional file 2: Figure S2.

## Results

### Overview of analyzed samples and results

We used clonality data from 60 samples derived from cross-sectional and longitudinal analyses of HTLV-1-infected individuals. Longitudinal monitoring of 21 cases (51 samples) was performed over a period of 15–90 months. Complete patient and sample information is provided in Additional file 1: Table S1. We followed each individual over time to capture the dynamics of clonality based on clone size and disease stage. Additional file 3: Table S2 summarizes the status of analyzed samples.

In total, nine ACs (individuals 1–9; in Additional file 1: Table S1) were analyzed at two or more time points. Five retained their AC status (individuals 1–5), whereas four progressed to ATL (individuals 6–9). All individuals who remained ACs had combinations of S and VS clones (polyclonal pattern; for clone sizes, see Additional file 1: Table S1). The size of the largest clone in individuals 1–5 at time point 1 (samples H-3, H-9, H-17, H-2, H-8) was 46, 357, 105, 77, and 388 cells, respectively, and at time point 2 (samples H-1, H-7, H-29, H-4, H-5) was 112, 433, 153, 187, and 244 cells, respectively. All samples from progressed ACs harbored B or VB clones at each analyzed time point.

In addition, 11 individuals with indolent types of ATL (smoldering (SM) or chronic) at time point 1 were analyzed, along with follow-up data at time point 2. Seven patients (individuals 10–16) were SM at time point 1. Of these, five (individuals 11–15) progressed to the chronic subtype at time point 2, one (individual 16) progressed to the acute subtype, and one (individual 10) remained SM. Four patients (individuals 17–20) were in the chronic state at time point 1. Of these, three (individuals 17–19) remained chronic and one (individual 20) progressed to the acute state at time point 2. Individual 21 was an acute patient who was monitored at 4 time points before, during, and after therapy. Finally, clonality data of some cross-sectional samples (individual 22–29) were also included.

### Is there a correlation between clone size and number of integration sites?

**Fig. 2:**
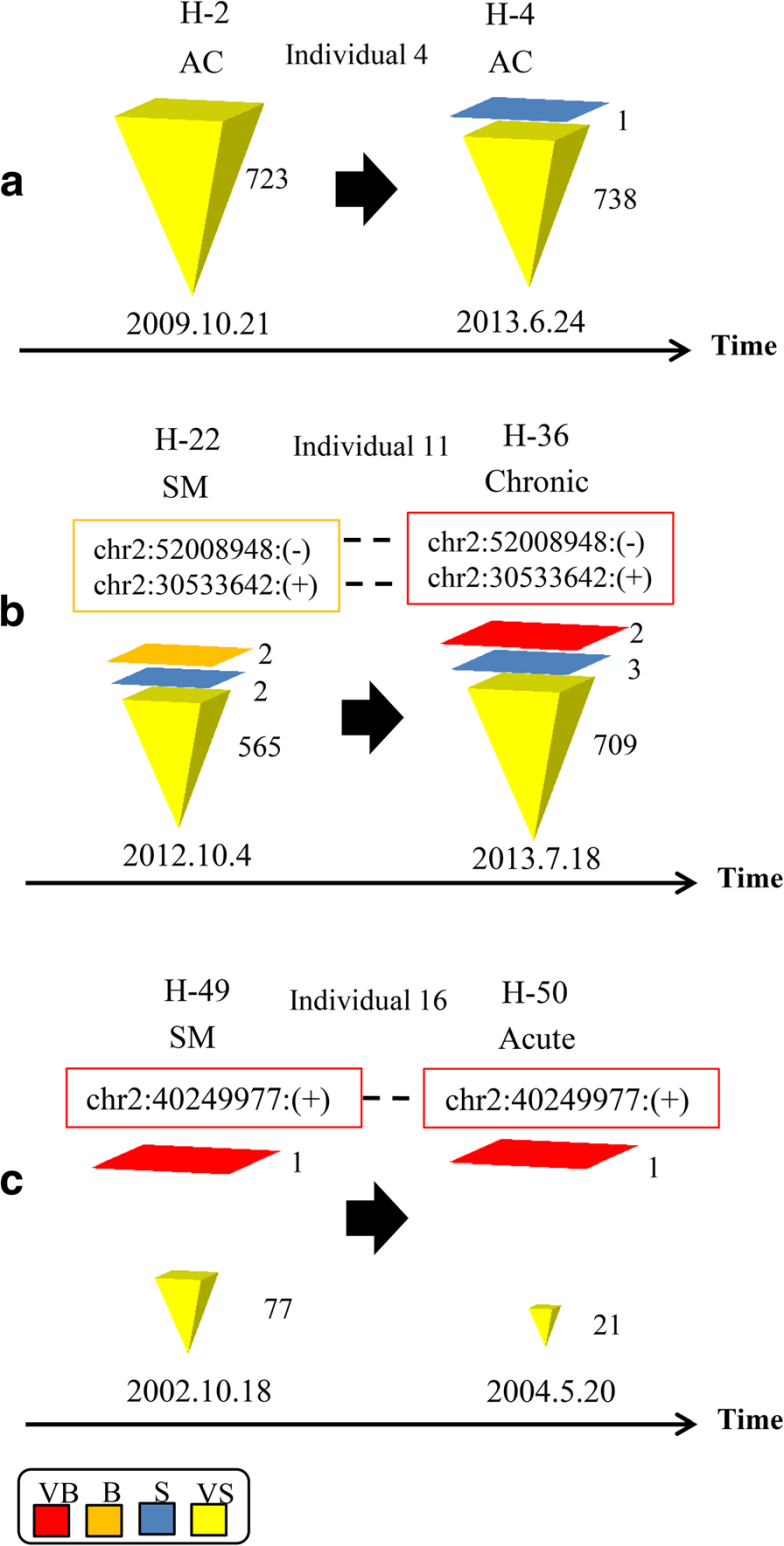
Clone size and integration sites in three representative individuals. Individuals are indicated at the *top*, followed by sample numbers and disease states at times 1 and 2. The number of integration sites is indicated beside color-coded representations of clone size. Clones with identical integration sites are shown in *boxes* color-coded for clone size and connected by *dashed lines*. **a** Individual 4 remained an asymptomatic carrier (AC) over time and had a large number of integration sites that fluctuated over time. **b** Individual 11 progressed from smoldering (SM) to chronic ATL. The integration sites of the two largest clones were identical over time. **c** Individual 16 progressed from SM to acute ATL. The integration site of the largest clone was identical over time. Results for other samples are shown in Additional file 2: Figure S3

**Fig. 3:**
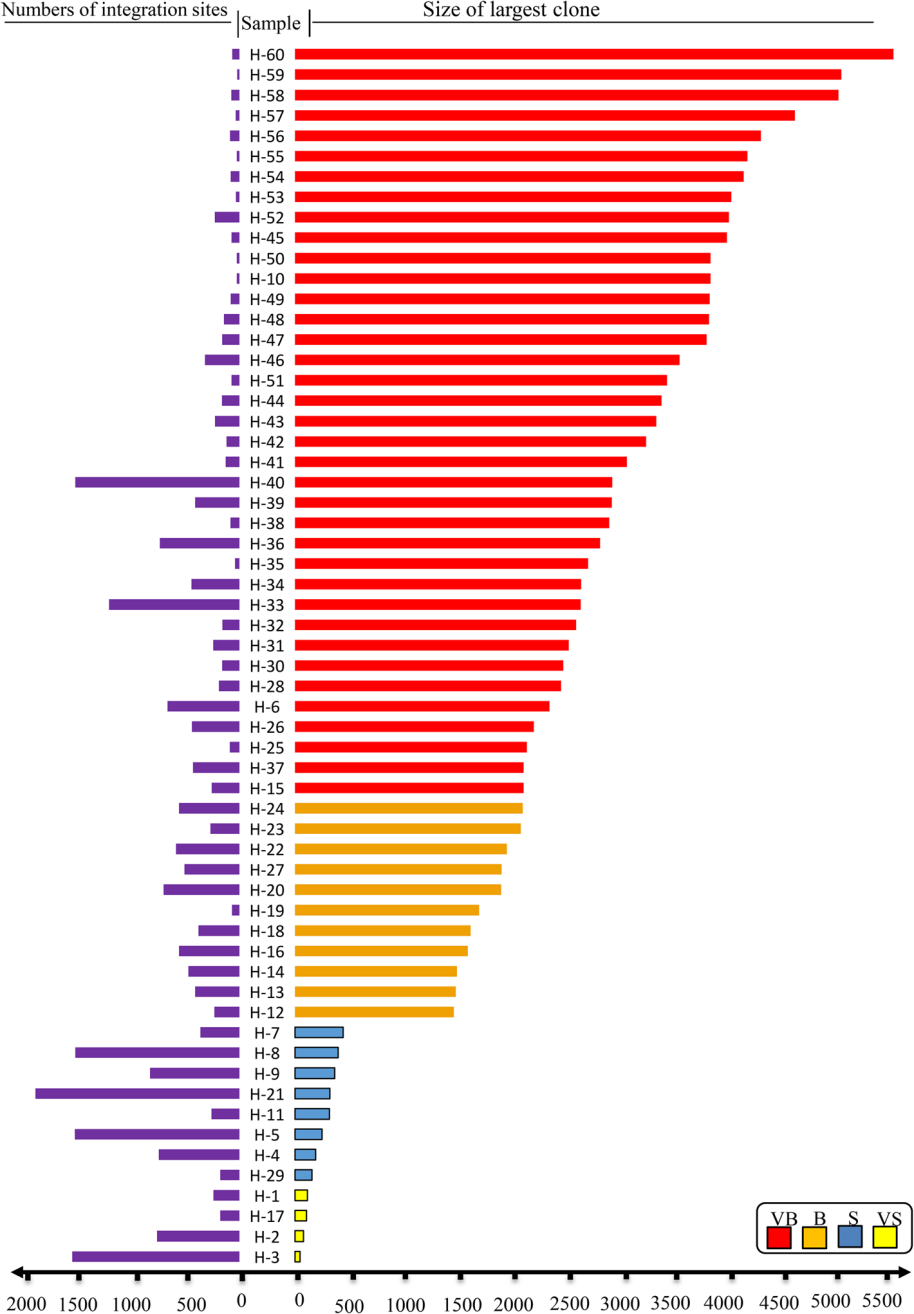
Correlation between number of integration sites and the size of the largest clone across all samples. A moderate negative correlation was observed (*R* = –0.55 based on Pearson correlation coefficient)

**Fig. 4:**
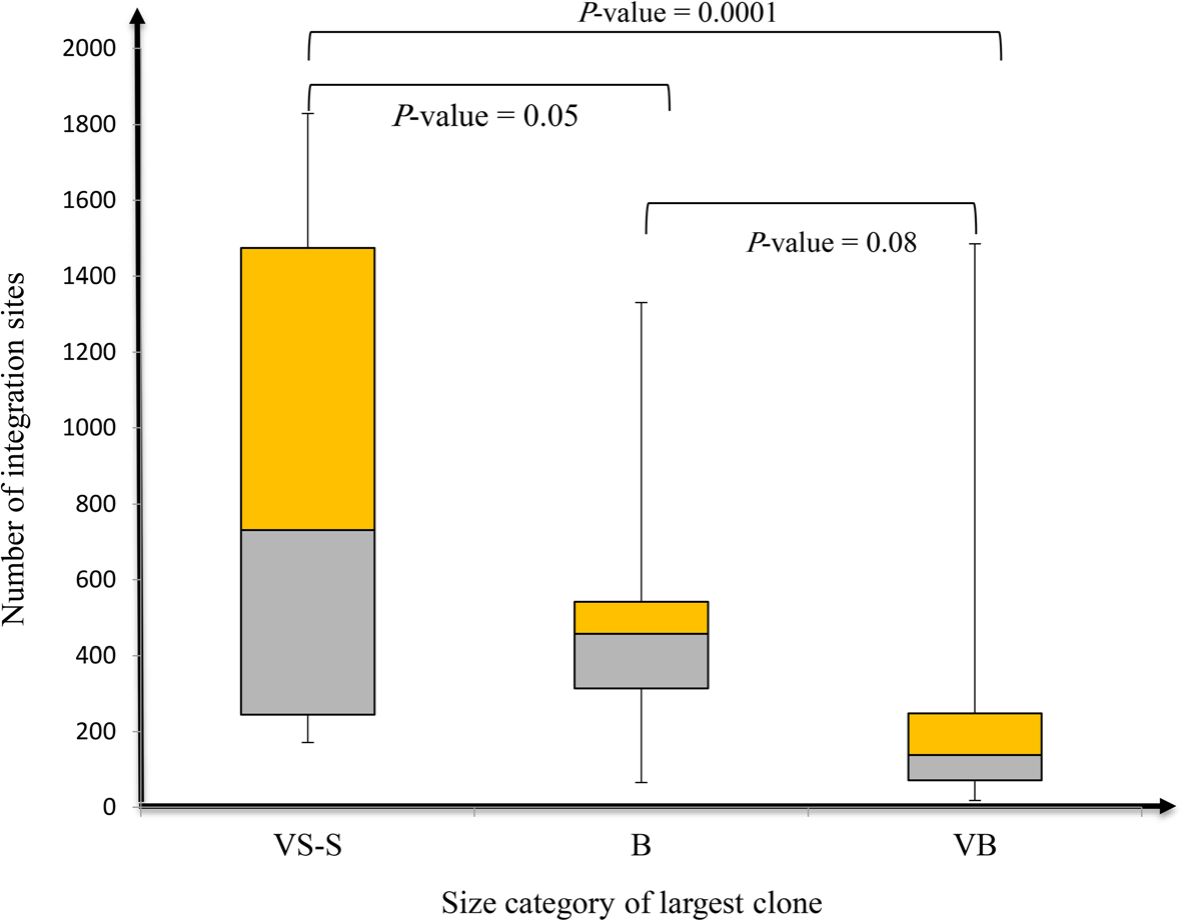
Number of clones belonging to size categories across analyzed samples. Samples were divided into three groups (VS-S, B, and VB) based on the category of the largest clone. The number of integration sites isolated from each group was then plotted. *P* values were calculated by Student’s *t* test

The maximum and minimum numbers of isolated integration sites across samples were 1832 and 18, respectively. The maximum and minimum sizes of the largest clones across samples were 5377 and 46, respectively. Figure 2 illustrates the number of integration sites belonging to different clone size categories (VB, B, S, VS) in three representative individuals monitored over time. In Fig. 3, we plotted the largest clone size and number of integration sites isolated from all samples. The Pearson correlation coefficient (*R* = –0.55) indicates a moderate negative correlation. Box plots in Fig. 4 illustrate the relationship between the number of integration sites and the size of the largest clone in an individual; the samples were divided into three classes, VS-S, B, and VB. Most samples with larger clone sizes had fewer integration sites. The differences in the number of sites between the VS-S and B categories and between the VS-S and VB categories were significant (*P* = 0.05 and *P* = 0.0001, respectively; Fig. 4). The *P* value of the difference between B and VB categories was 0.08 (Fig. 4). Samples with larger clones had significantly fewer integration sites (Fig. 4). An exception to this trend was seen in individual 13 (samples H-40 and H-33 in Additional file 1: Table S1 and Additional file 2: Figure S3). This patient was SM at time point 1, with one VB, one S, and 1472 VS clones (H-40) and progressed to chronic at time point 2, with one VB, one S, and 1169 VS clones (H-33). Although the biological reason for the atypically large number of VS clones in this patient is unknown, this observation suggests that there is a low probability of a technical limitation in detecting background S and VS clones in the presence of B and VB clones. Thus, the presence of large clones and a small numbers of S and VS clones might be of prognostic value.

**Fig. 5:**
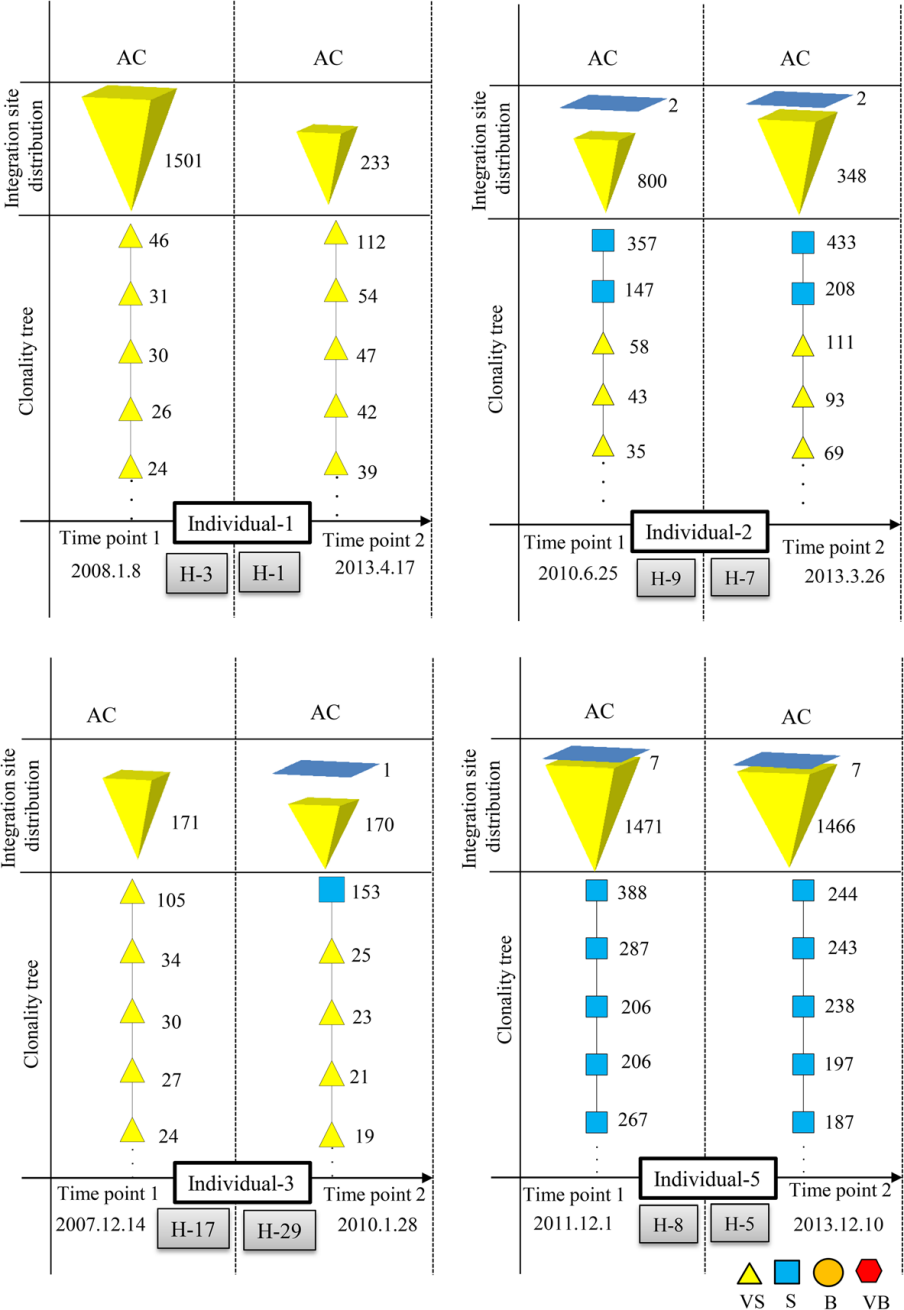
Clonality data of ACs who remained ACs. High numbers of integration sites were detected from each sample. Only S and VS clones were observed

**Fig. 6:**
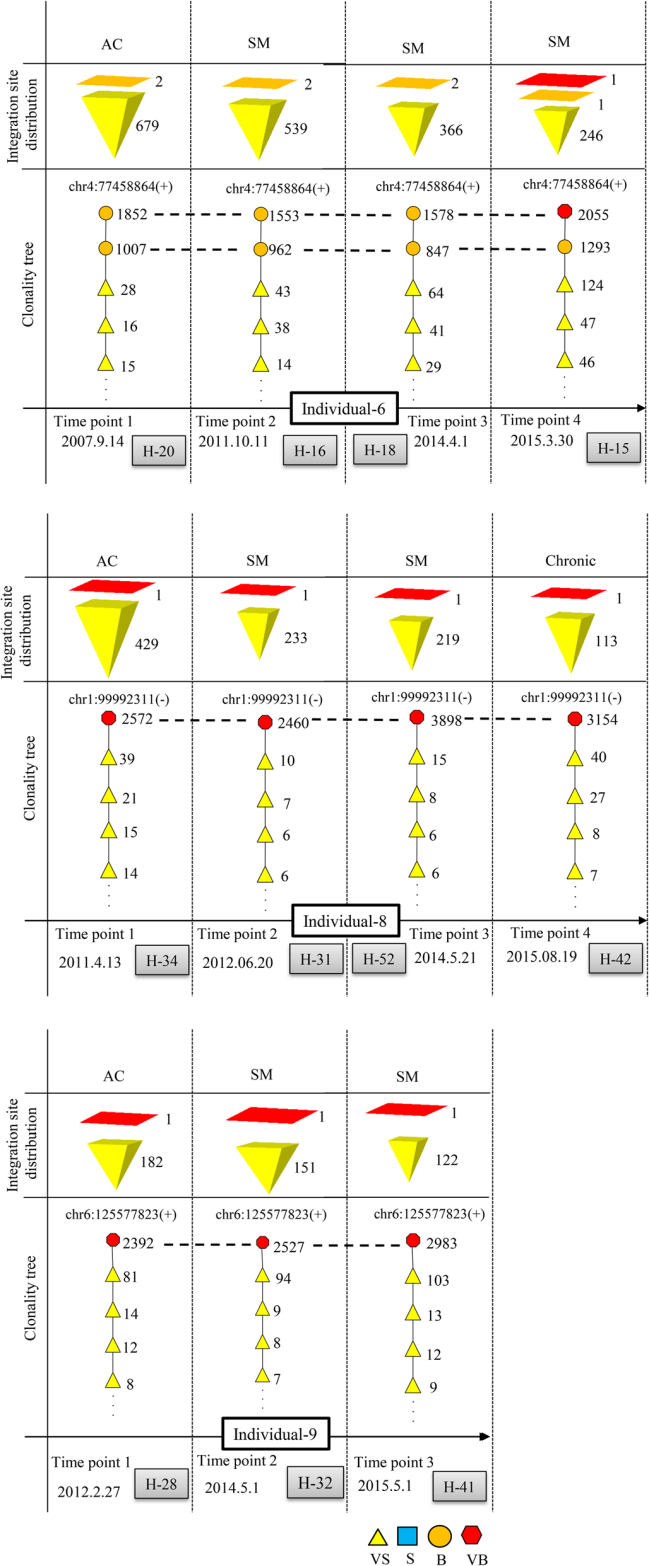
Clonality data of ACs who progressed to ATL. Clones with identical integration sites are connected by *horizontal dashed lines*. B or VB clones were detected in addition to VS and/or S clones, and their integration sites were constant over time

ACs who remained ACs over time had only combinations of VS and S clones (Figs. 5 and 2a). In contrast, ACs who progressed to different subtypes of ATL had B or VB clones in addition to VS or S clones. The B and VB clones in ACs at early time points were also detected during the malignant stage of the disease (Fig. 6 and Additional file 2: Figure S3). All observed clonality patterns for cross-sectional samples are shown in Additional file 2: Figure S4. Only the five largest clones in each sample are shown because significant changes in clone size occur only at this level. Below this level, only S and VS clones are observed.

### An informative way to interpret and visualize data

After processing high-throughput clonality data and measuring clone sizes for each sample, we ordered clones based on size in a descending manner and categorized them into VB, B, S, or VS groups. Subsequently, we represented the clonal structure of ACs and different subtypes of ATL using different shapes and colors.

To reflect clonality behaviors and prognosis status of HTLV-1–infected individuals, we modeled the observed data using the concept of a rooted ordered max-tree and m-ary (1 ≤ *m* ≤ 4) tree (Additional file 2: Figure S2). The tree vertices (V) are defined by the four clone size categories: *V* = {VB, B, S, VS}. The tree edges (E) have ten possible members *E* = {VB-VB, VB-B, VB-S, VB-VS, B-B, B-S, B-VS, S-S, S-VS, VS-VS}. In the proposed tree structures, the key values indicate clone size. To discriminate and visualize clone sizes, different shapes and colors are used. We first provided four basic ordered max-trees based on clone size as primary rules (Additional file 2: Figure S2B). We then constructed tree structures for the clonality pattern of each sample using these four basic trees that are ordered based on clone size (Additional file 2: Figure S2C). These trees represent the relationship between the clone sizes in each analyzed sample. We defined a path in the proposed trees as a set of edges from an ancestor node to a specific leaf of that tree (i.e., to a terminal node). In the proposed hierarchical tree model, each node of a tree corresponds to a unique observed clone. The edges between vertices and the order of vertices are designed based on the clone size. Each path represents the clonality status of a sample, and clone size is seen to decrease along the path.

**Fig. 7:**
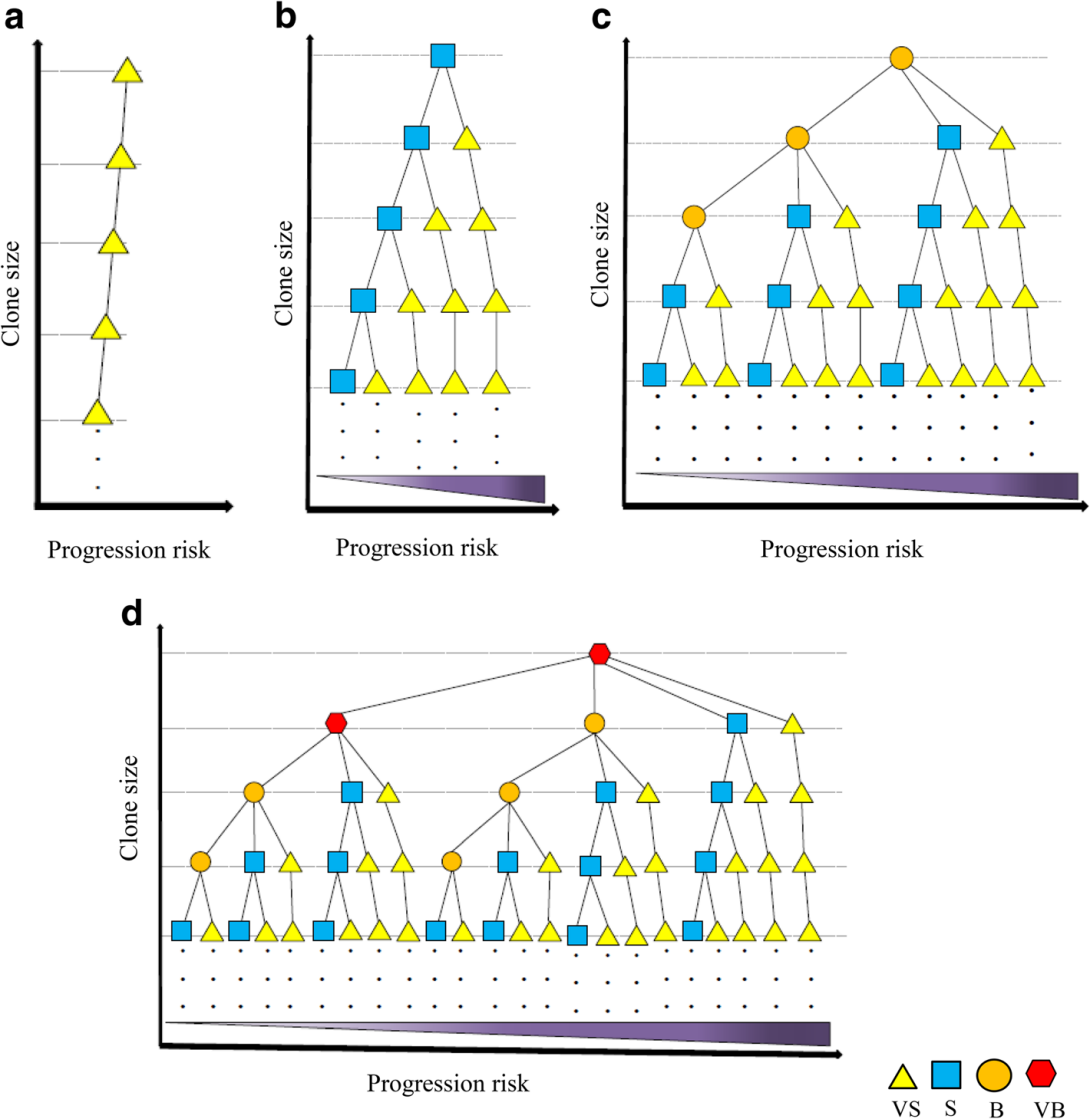
**a**–**d** Global scheme of clonality dynamics among analyzed samples represented by tree structures. Each tree begins with a root of a different clone size (VS, S, B, and VB). The clonality status of each sample can be presented by an individual path. Within each tree, clone size decreases from the root to the leaves. In addition, samples on the right side of a tree have a greater risk of disease progression compared with those on the left. Thus, clone size, order, and disease progression of each sample can be compared to the other samples

In Fig. 7, we augmented this sample-by-sample view by including disease progression, which increases from left to right as the disease approaches full malignancy and the prognosis worsens. This hierarchical structure presents clones from each sample based on the size, order, and disease progression stage, allowing comparisons with other samples (Fig. 7). This illustration can be used as a guide for predicting prognosis based on clonality among infected individuals. Importantly, to construct these final trees and determine the order of paths, we used real clonality data obtained from our cross-sectional and longitudinal analysis. It is noteworthy that no sample had more than two VB clones and three B clones. Also, the number of S clones was limited as compared with that of VS clones, which were much more numerous. VB clones should be located at level 0 and/or 1, and B clones can be observed up to level 3 (4th level of tree). In principle, nodes can be shown for any number of levels (as indicated by dots in Fig. 7). Here, we used only five levels because our data indicate that diversity in clone sizes is observable only up to the 4th level, after which only S and/or VS clones are detectable. The number of vertices in m-ary trees theoretically is calculated from Eqs. 1 and 2 (Additional file 2: Figure S2). The actual number of observed vertices based on observed clonality patterns is much less than full m-ary tree because of the pruning of some edges. The proposed tree was validated by the clonality of all 60 analyzed samples.

### Representative examples to intuitively depict how the model works

Trees of all samples without progression from AC (individuals 1–5) are represented in Fig. 7a, b. All other samples (patients with progression to different subtypes of ATL) are represented in Fig. 7c, d. The order of paths in each panel is determined based on disease progression. For example, trees of individual 15 belong to Fig. 7c. This patient was SM at time point 1 (H-14), and the tree was located to the left; at time point 2 (H-23), following progression to the chronic state, the tree moved to the right (Additional file 2: Figure S3). Trees of all acute individuals belong to the right side of Fig. 7d. Samples with a single B or VB clone had worse prognoses than those with two or more, and thus, their paths were located toward the right.

**Fig. 8:**
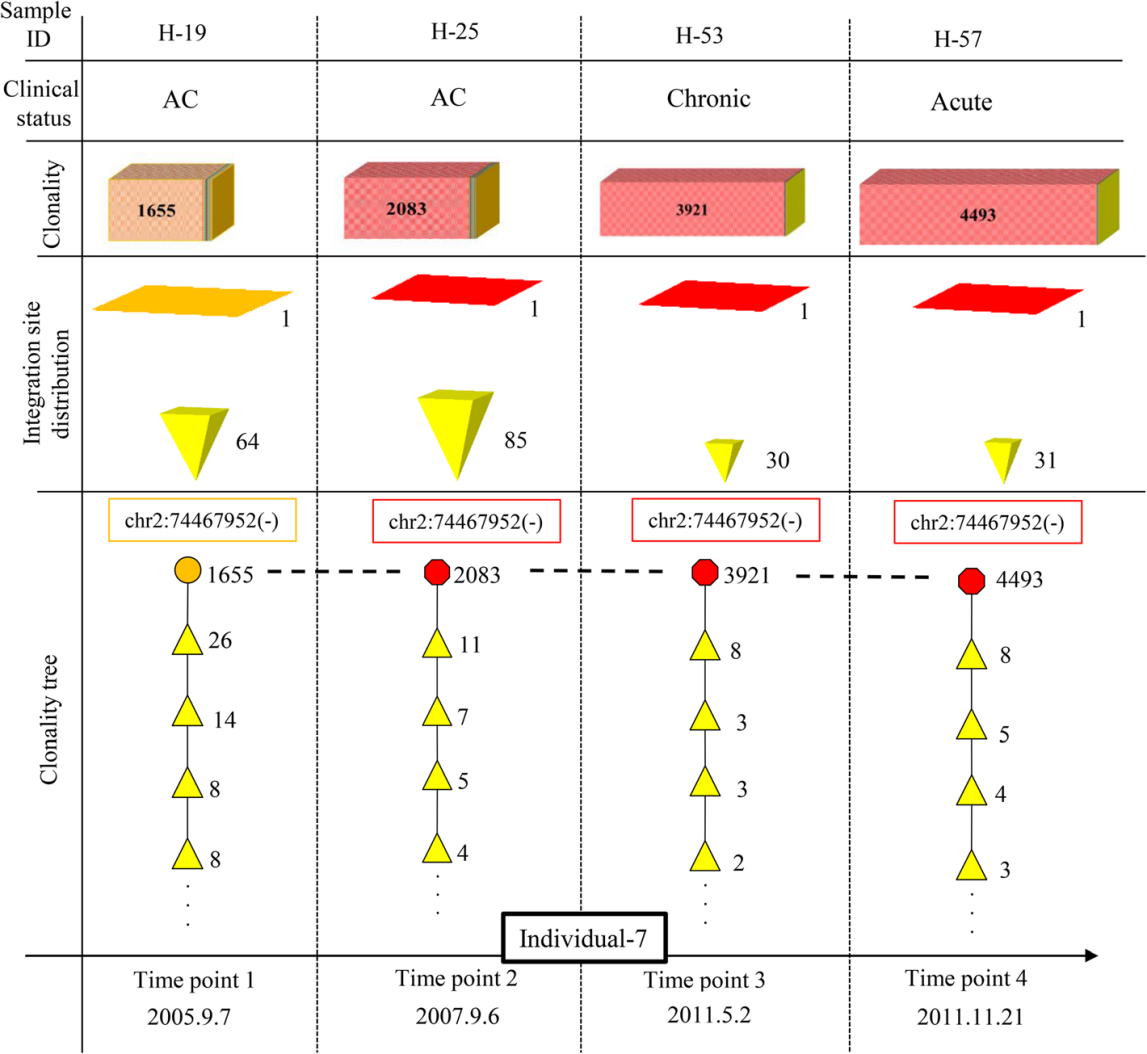
Clonality tree for a patient (individual 7) monitored over 6 years who progressed from AC status to ATL onset. The patient had a B clone at time point 1 and a VB clone at time points 2, 3, and 4. The integration site of the largest clone was identical across all time points, as indicated by the *dashed horizontal lines*. Clonality of this high-risk sample was notably different from that of a typical AC

As an example, we present the case of individual 7, who was monitored at four time points over 6 years (Fig. 8). Unlike typical ACs, who manifest a combination of S and/or VS clones (polyclonal pattern), this individual showed largely expanded clones, with one B clone at time point 1 (H-19) and one VB clone at time point 2 (H-25). Therefore, the path for the clonality of this sample was located on the right side of the global tree (Fig. 7c), which indicates a higher risk of ATL development in this sample. Consistent with our prediction from the model, this individual subsequently progressed to the chronic state at time point 3 and the acute state at time point 4. Clonality trees for the remaining samples are presented in Figs. 5 and 6 and Additional file 2: Figures S3 and S4. As noted above, individuals 1–5 harbored only S and VS clones and remained as ACs over time; their clonality trees were located on the left side of the global tree, which is considered very low risk (Fig. 5).

Individual 6 was an invaluable AC, with two atypically large B clones at time point 1. Therefore, the clonality tree was located in a higher risk area. Consistent with the model, the patient developed SM ATL at time point 2 (H-16) and remained SM at time point 3 (H-18). At time point 4 (H-15), this patient showed a VB clone, a B clone, and an increased proviral load (PVL) (Fig. 6). Individual 8 was an atypical AC with a VB clone at time point 1 (H-34). This individual progressed to SM at time point 2 (H-31), remained SM at time point 3 (H-52) and finally progressed to chronic state at time point 4 (H-42). Individual 9 was a rare AC with an atypical VB clone at time point 1 (H-28), and thus, the clonality tree was located in a higher risk area. This individual progressed into SM ATL at time point 2 (H-32) and remained SM at time point 3 (H-41). Ultimately, this patient developed acute ATL and died of the disease, but clonality data at this stage were not available (Fig. 6). The clonality paths for all acute samples were located at the right side of the global tree (Additional file 2: Figure S3).

Another interesting case was an acute patient (individual 21) who was monitored at four time points before, during, and after treatment. The absolute sizes of the largest clone at each time point were 2848, 2411, 2147, and 4066 cells, and the integration site was located at chr9:22270886 (–). This clone belonged to the VB group; the patient remained resistant to therapy and ultimately relapsed. The number of background VS and S clones was H-30: 152 VS; H-39: 394 VS, 2 S; H-26: 420 VS, 4 S, and H-55: 21 VS. The number of integration sites fluctuated during therapy but was greatly reduced after relapse (Additional file 2: Figure S3).

## Discussion

Cancer is a complex disease characterized by many different traits, making it an ideal target for various modeling approaches [28]. Well-designed visualization models can transform raw data into logically structured and visually informative representations capable of revealing patterns and structures that otherwise remain hidden [45]. Motivated by the increased availability of NGS data on clonality, we focused on the problem of constructing a progression model of ATL. Development of such progression models is very important for predicting prognosis and, hence, for timely therapeutic decision-making. We focused on clonal expansion as a hallmark of ATL and provide an overall picture of clonality using intra- and inter-sample clinical data.

HTLV-1-infected individuals have an about 3–5% lifetime risk of developing ATL [11]. Currently, there is no established method to clearly predict the risk of progression to ATL. The current risk indicator is PVL; however, individual patient prognosis based on PVL alone is difficult because the range of PVLs among patients with disease overlaps that of patients who remain lifelong ACs [52–54]. Therefore, additional prognosis indicators are required. It is known that clonal proliferation of infected cells is primarily responsible for maintaining the PVL of HTLV-1 [55]. Thus, investigating the clonal composition of HTLV-1-infected individuals as a potential risk indicator of ATL would be of great clinical and biological significance. Understanding how clonal expansion occurs could be used as a key factor in finding effective ways for early detection of ATL, enabling more timely and precise treatment. To address this problem, we converted complex clonal expansion data into a manageable model of relationships among a set of clones that incorporates the characteristics of clone sizes, clone order, and disease progression over time. Using this approach, we were able to provide answers to several important questions [12, 26, 56, 57]:

1. What is the role of clone size and combinations in ATL progression?

Only ACs with B or VB clones progressed to ATL [42]. Samples with a single B or VB clone had worse prognoses than those with two or more, and thus, their paths were located toward the right of the proposed model. All individuals who progressed to higher states of ATL harbored B and/or VB clones. The presence of VB and B clones, as well as small numbers of S and VS clones might be of prognostic value. (Figs. 5 and 6, Additional file 2: Table S2, Fig. 7).

2. How many distinct HTLV-1^+^ clones of various sizes are detectable from a single host?

We detected a maximum of two VB clones, three B clones, a small numbers of S clones, and a large number of VS clones.

3. What is the relationship between clonal composition and the risk of ATL onset?

Based on the observed clonality data and clinical status of each individual, we ordered the observed trees of each sample and proposed a global tree model. Samples for which trees were located at the left side of the global tree had less risk of disease development, whereas those located at the right side had higher risks (Fig. 7).

Our longitudinal data provided further direct evidence that HTLV-1 can be integrated and persist in humans for years. We also confirmed that each HTLV-1-infected individual carries a large number of infected clones of different sizes. S and VS clones were much more numerous than B and VB clones. We showed that selective clonal expansion or survival of certain transduced or infected cell clones is a limited occurrence, as no more than two VB clones and three B clones was detected in any individual. Moreover, only certain clones that were already dominant (B/VB) at a non-malignant state gave rise to dominant clones during the malignant disease. None of the samples with only S and VS clones belonged to patients who developed ATL over time. Thus, consistent with what we have reported previously [42], the clonality of HTLV-1-infected cells could be a useful predictive marker of ATL onset and progression.

Combining biological experimental data with computational analysis and mathematical modeling facilitates quantification and accurate description of biological phenomena. Using simpler mathematics is recommended to improve communication with experimental biologists [58]. In general, understanding analytical deviations, complicated mathematical equations and formalisms is difficult for the average biologist. It has been suggested that a large number of equations in a paper is associated with a reduced impact of the study among biologists [58, 59]. Therefore, strong and complicated technical presentation of mathematical models may reduce their impact and comprehension among biologists. Moreover, avoiding an overdependence on theory and integrating experimental data are important and appropriate strategies that enhance the effectiveness of models [32]. Bridging analysis and visualization of data—particularly omics data—not only facilitates the presentation of information but also may uncover unknown concepts, conclusions, and insights about the results.

The model of ATL clonal expansion presented here permits investigation of the impact of clonal expansion and related parameters on the risk of ATL development by offering a new understanding of how clonality patterns contribute to disease progression. This easy-to-understand model is an appropriate data-driven model for ATL clonality because it uses real biological data without theoretical assumptions and relies on a small number of variables and a simple set of relationships to explain the clonality status of samples.

## Conclusions

Here, we used tree-based structures to show clonality patterns and to display the importance of clone size and clonal composition in ATL development. This integration site-based clonality tree model (1) has both descriptive and prognostic capacities, (2) provides clear visualization of the results, (3) uses simple mathematics, and (4) is fully data-driven and is not dependent on theoretical assumptions. The proposed hierarchical tree model will be useful for organizing and summarizing a mechanistic understanding of observed results and for providing significant insights into clonal history based on the integration sites of HTLV-1-infected cells. To our knowledge, this is the first model that enables the prediction of prognosis among infected individuals based on clonality information (clone size and dynamics of clones). Designing a scoring system for representing the clonal expansion process and implementing a user-friendly software tool for clinical applications are of high interest for future studies.

## Additional files

Additional file 1: Table S1. Sample identification and summary of data. (XLSX 15 kb)

Additional file 2: Figure S1. Quality control of sequencing output. **Figure S2.** Trees that can represent hierarchical clonal structures based on clone size. **Figure S3.** Clonality data for longitudinal samples. **Figure S4.** Clonality data for cross-sectional samples. (PDF 1247 kb)

Additional file 3: Table S2. Disease status and clonal analysis over time. (PDF 234 kb)

## Abbreviations

AC: Asymptomatic carrier
ATL: Adult T cell leukemia
B: Big
HTLV-1: Human T cell leukemia virus type 1
JSPFAD: Joint Study on Predisposing Factors of ATL Development
NGS: Next-generation sequencing
PVL: Proviral load
S: Small
SM: Smoldering
VB: Very big
VS: Very small
