# Supplementary material for: Inferring clonal structure in HTLV-1-infected individuals: towards bridging the gap between analysis and visualization
Source: Hum Genomics. 2017 Jul 11;11:15. doi: 10.1186/s40246-017-0112-8 (PMC5505134; doi:10.1186/s40246-017-0112-8)
Supplement: Supplementary file 2 — Quality control of sequencing output. Figure S2. Trees that can represent hierarchical clonal structures based on clone size. Figure S3. Clonality data for longitudinal samples. Figure S4. Clonality data for cross-sectional samples. (PDF 1247 kb) [file 40246_2017_112_MOESM2_ESM.pdf]

Additional file 2

Inferring clonal structure in HTLV-1–infected individuals: towards bridging the gap between analysis and visualization

Amir Farmanbar[1,2*], Sanaz Firouzi[1* §], Wojciech Makałowski[3], Masako Iwanaga[4], Kaoru Uchimaru[1], Atae Utsunomiya[5], Toshiki Watanabe[1,4], Kenta Nakai[1,2 §]

a

Lane	% PF	# Reads	Raw clusters per lane (%)	≥ Q30 Bases (%)	Mean Quality Score
1	97.56	432,996,822	100	93.24	36.42
2	98.68	414,487,980	100	93.82	36.60
3	98.58	466,773,885	100	92.96	36.47
4	98.41	434,157,576	100	93.62	36.55

b

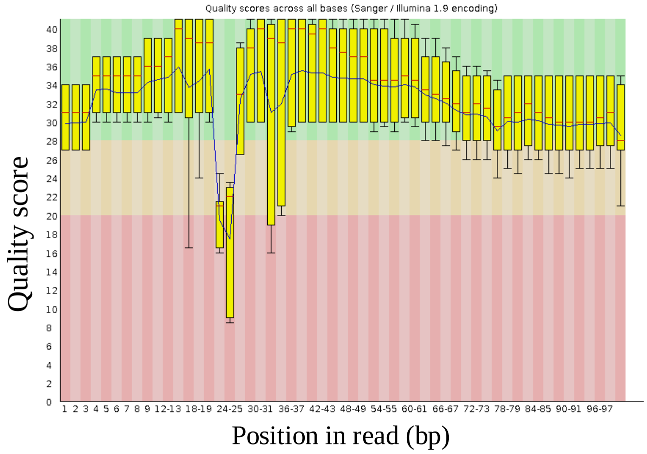

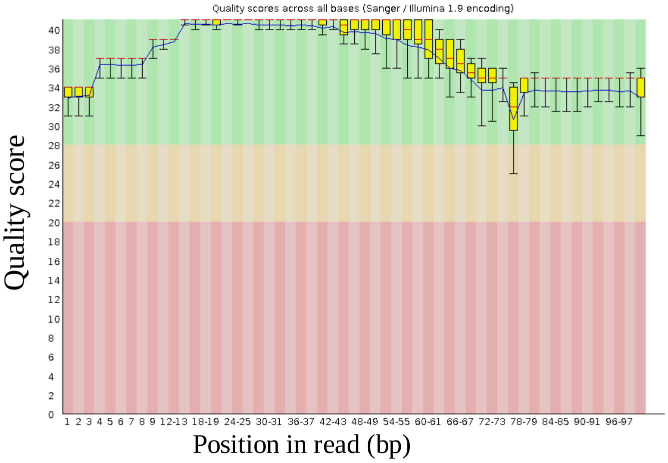

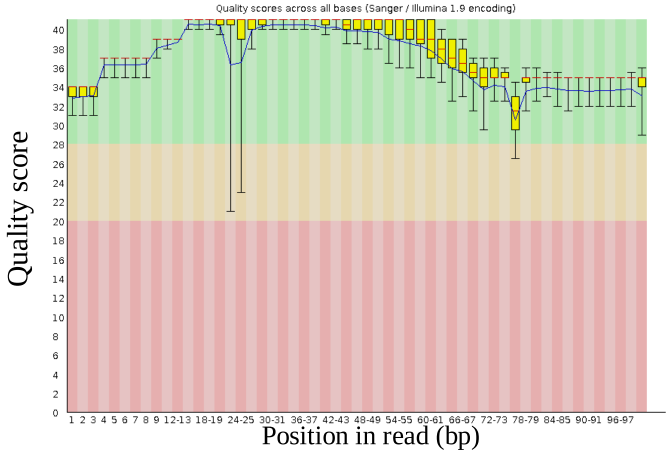

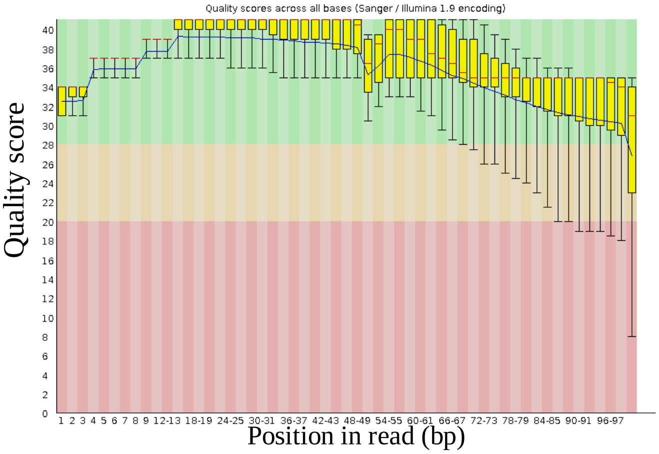

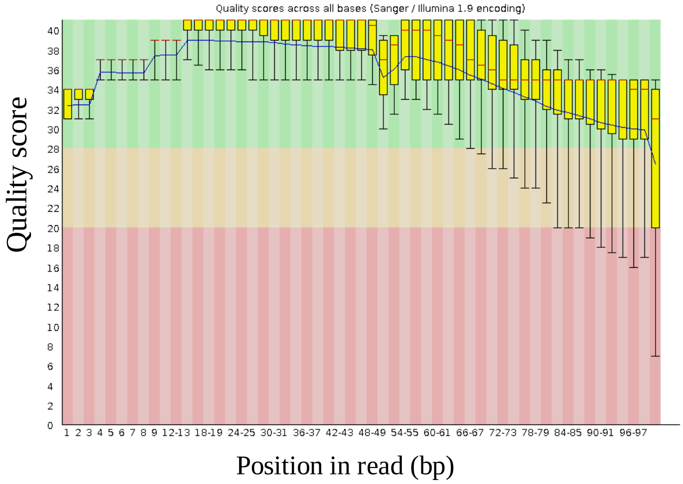

c

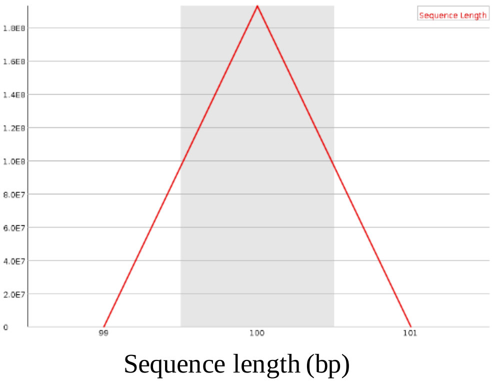

Figure S1. Quality control of sequencing output. a. Information on clusters passing filter (PF), number of reads, percentage of raw clusters per lane, percentage of reads with ≥Q30 bases (PF) and mean quality scores for four representative lanes of Illumina HiSeq 2500 sequencing data. b. Per-base quality control checks on raw sequence data from two lanes analyzed by FastQC (https://www.bioinformatics.babraham.ac.uk/projects/fastqc/). c. Sequence length distribution.

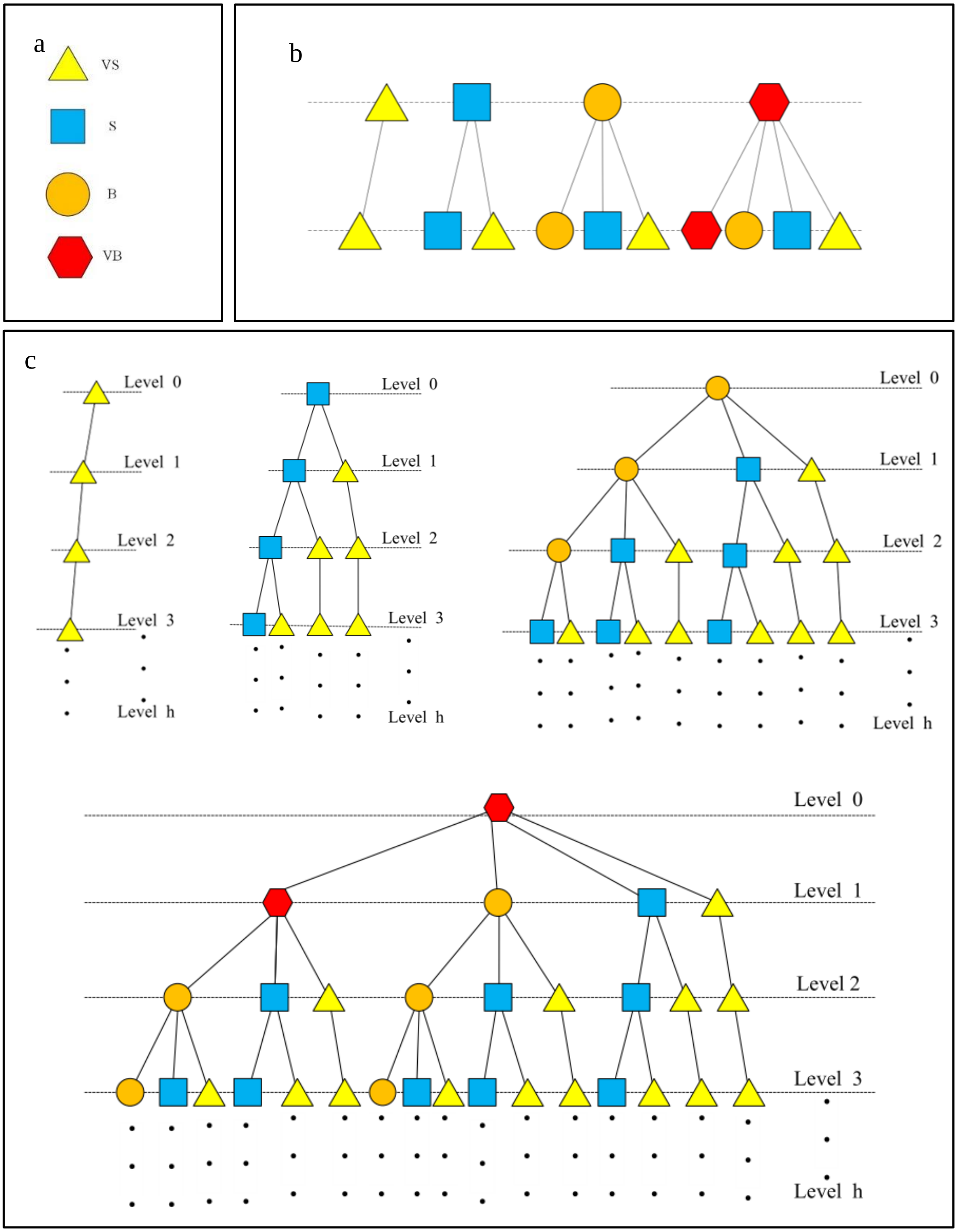

Figure S2. Trees that can represent hierarchical clonal structures based on clone size. a. Four symbols used to represent clone size (VS, S, B and VB). These are used as the nodes of trees. b. Basic ordered max-trees that can be generated by combinations of VS, S, B and VB clones. c. Possible ordered max-tree and m-tree structures extended up to four levels. The black dots indicate that the tree still has children.

Additional Information for Figure. S2:

The following definitions for tree structures were used in this study. A ‘tree’ is a subclass of graph theory [43, 44, 54] and can be formally defined as a connected graph (G) that does not contain cycles. It is represented by G = (V, E), where V refers to vertices and E represents the edges of G. Two nodes of a tree are connected via a single unidirectional path. Each node of a tree has one or more ‘children’ below it, but only a single ‘parent’ above it. Ancestors of a node are the ‘parent’, ‘grandparent’, ‘great-grandparent’, etc. A rooted tree is a tree in which there is one vertex that is distinguished from the others and is called the ‘root’. The root is the highest parent. ‘Leaves’ are nodes without children.

A ‘path’ in a tree is a set of edges from the root to a specific node. The height of a rooted tree is the maximum level of any vertex of the tree. An ordered tree is a rooted tree in which the children of each vertex are assigned a fixed ordering. We used m-ary and max-trees to model the observed data. In m-ary trees, the outdegree for every node is equal or less than (m). In other words, a rooted tree is called an m-ary tree if every internal vertex has no more than m children. In a max-tree, the value of each node is less than or equal to the value of its parent, with the maximum value at the root (parent ≥ child/children). In an m-ary tree: (1) maximum number of vertices at level $h \leq mh$, and (2) total number of vertices $\leq \sum_{k=0}^{h} m^k$

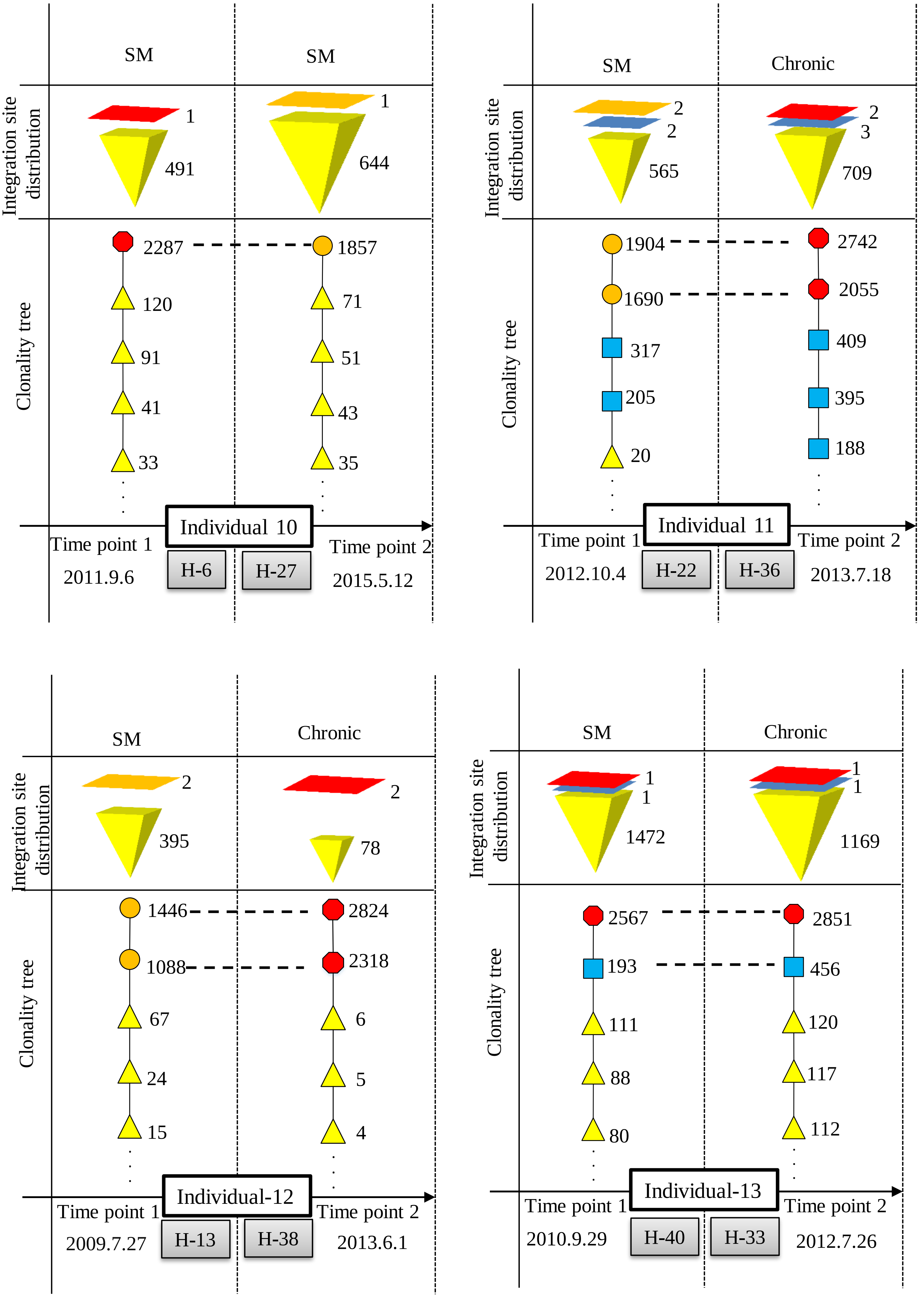

Figure S3. Clonality data for longitudinal samples.
Clones with identical integration sites are connected by horizontal dashed lines.

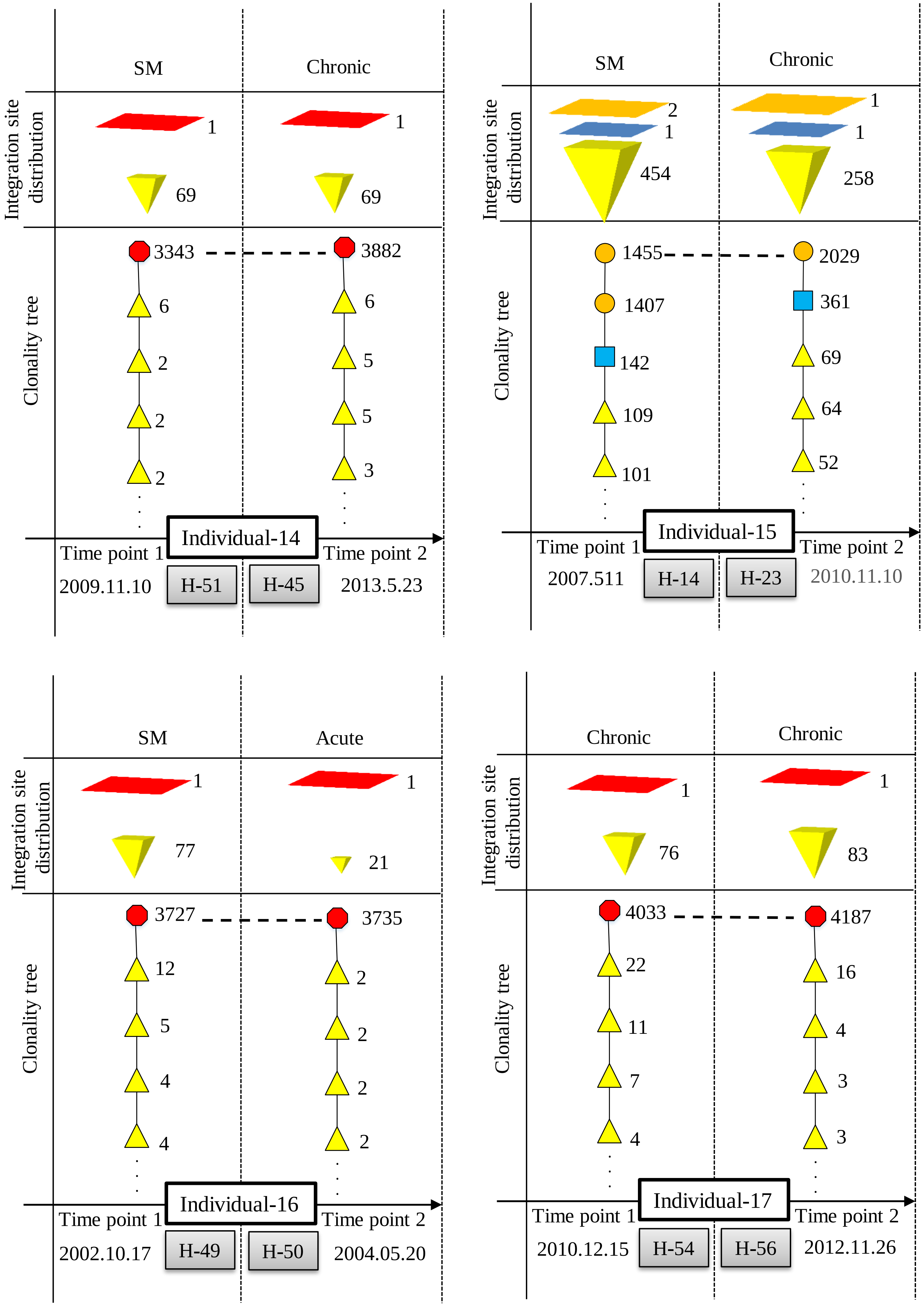

Figure S3. Continue

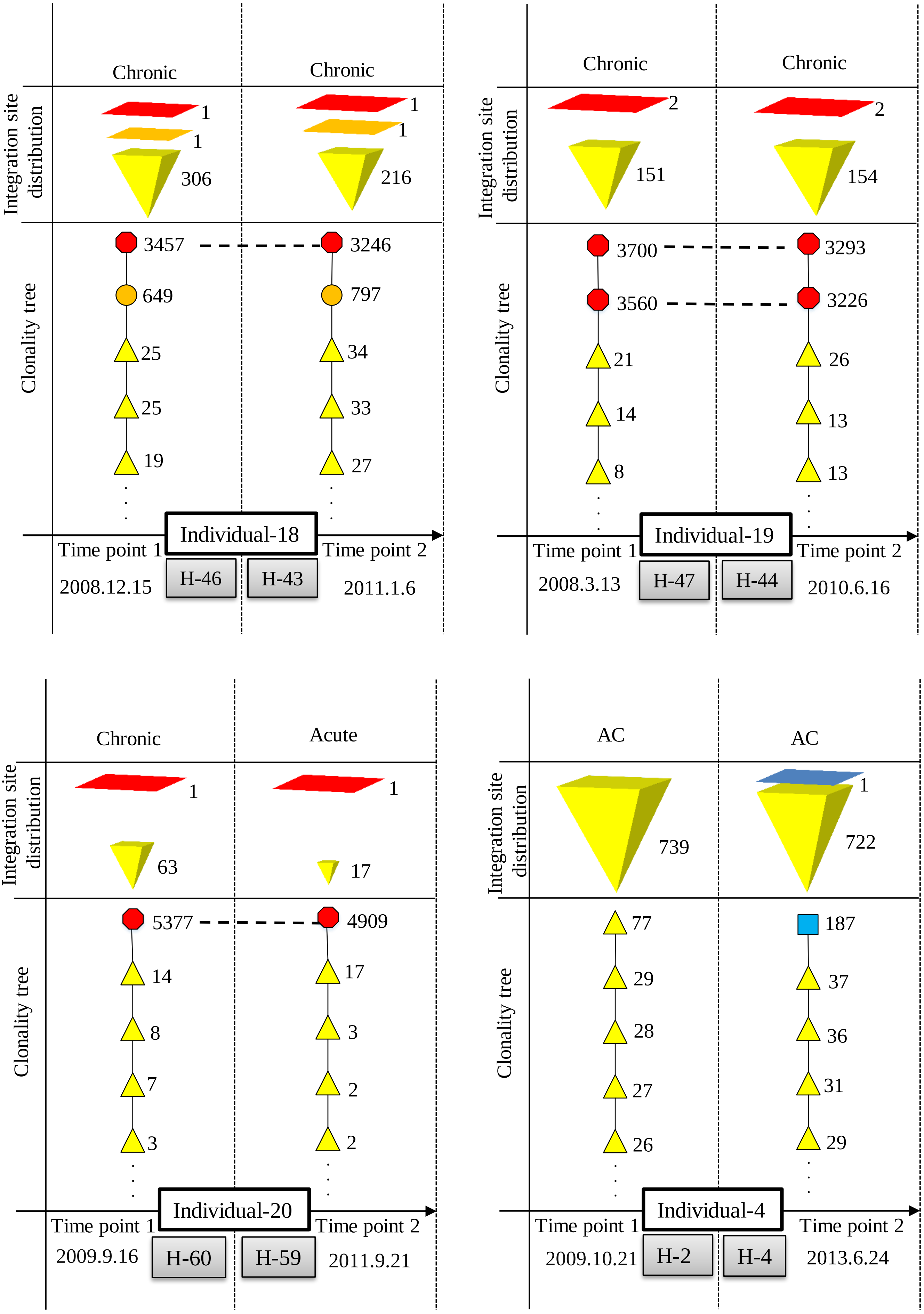

Figure S3. Continue

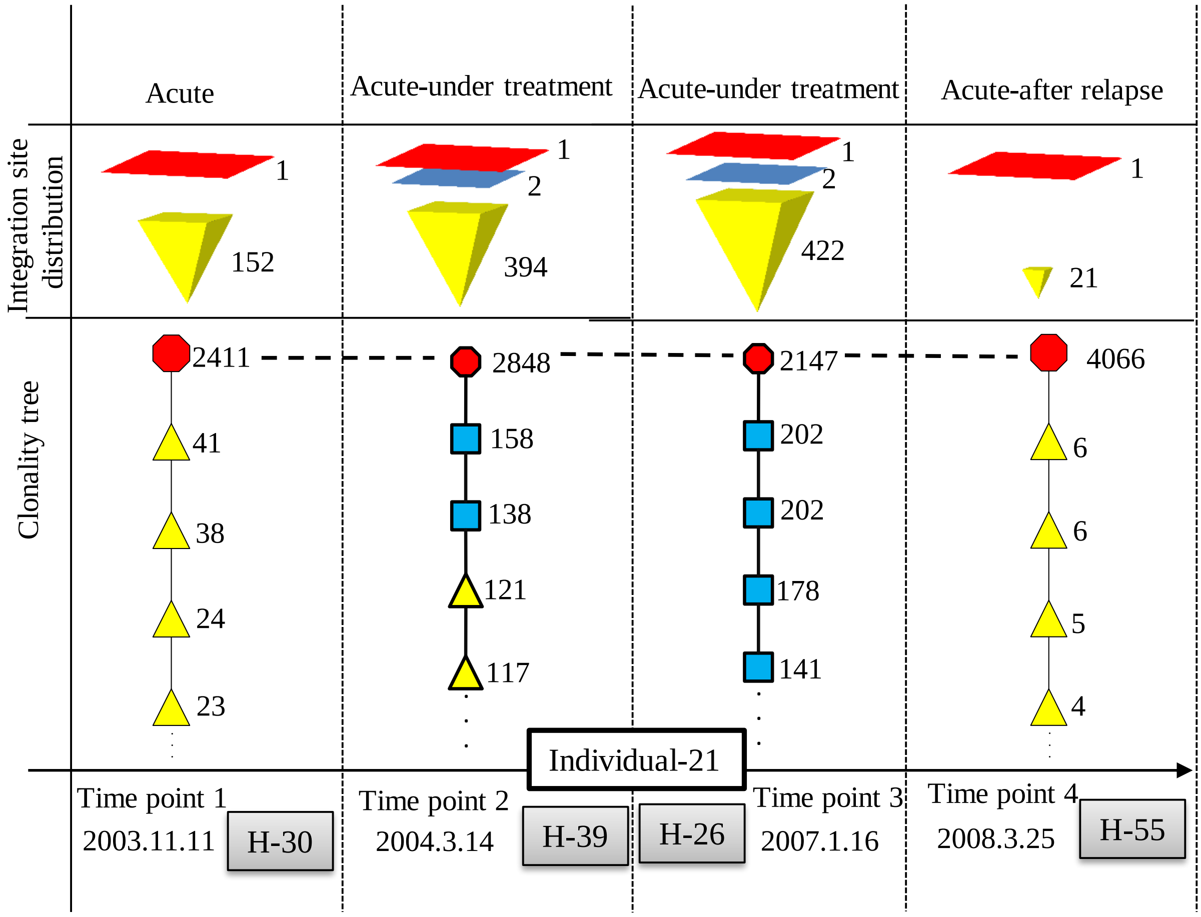

Figure S3. Continue

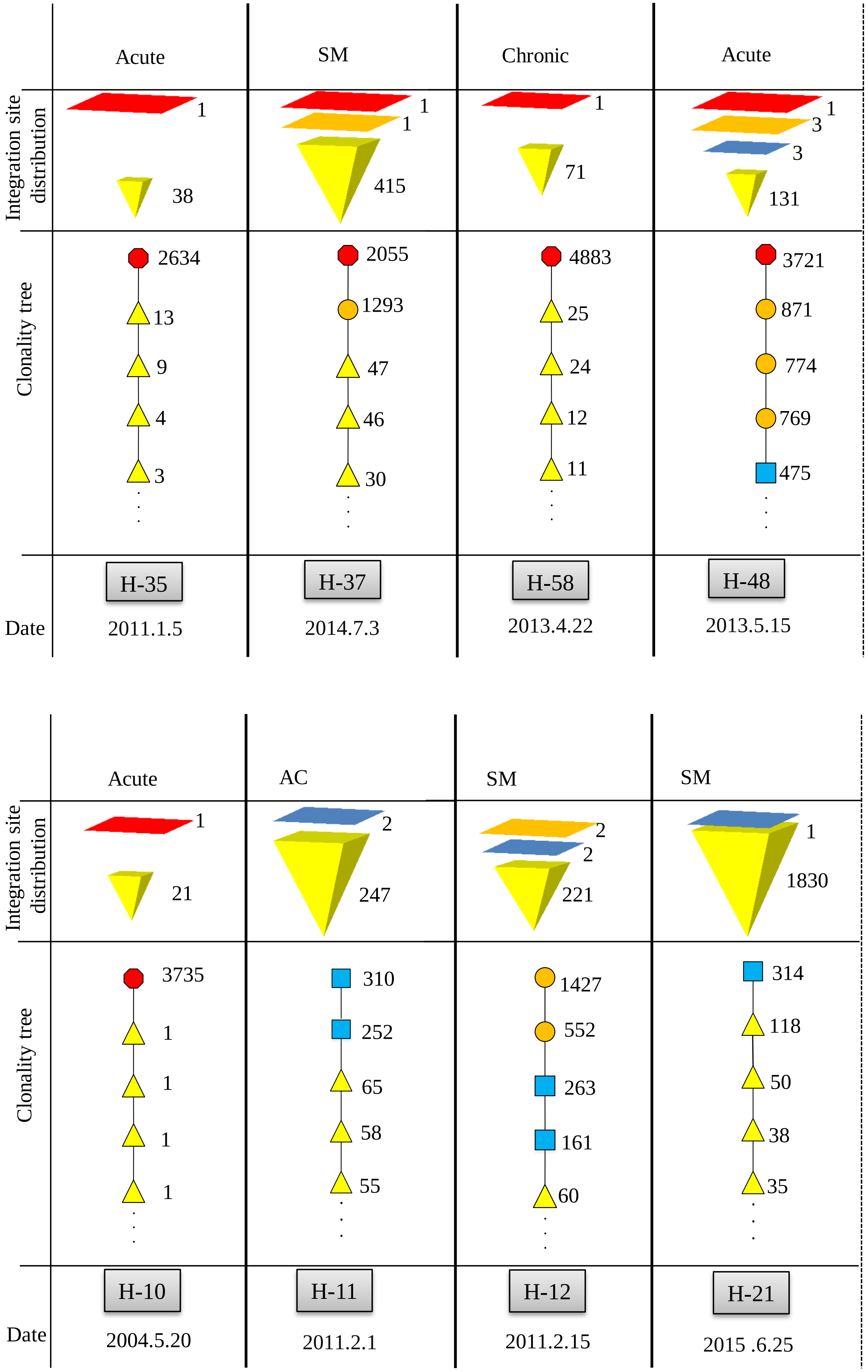

Figure S4. Clonality data for cross-sectional samples.

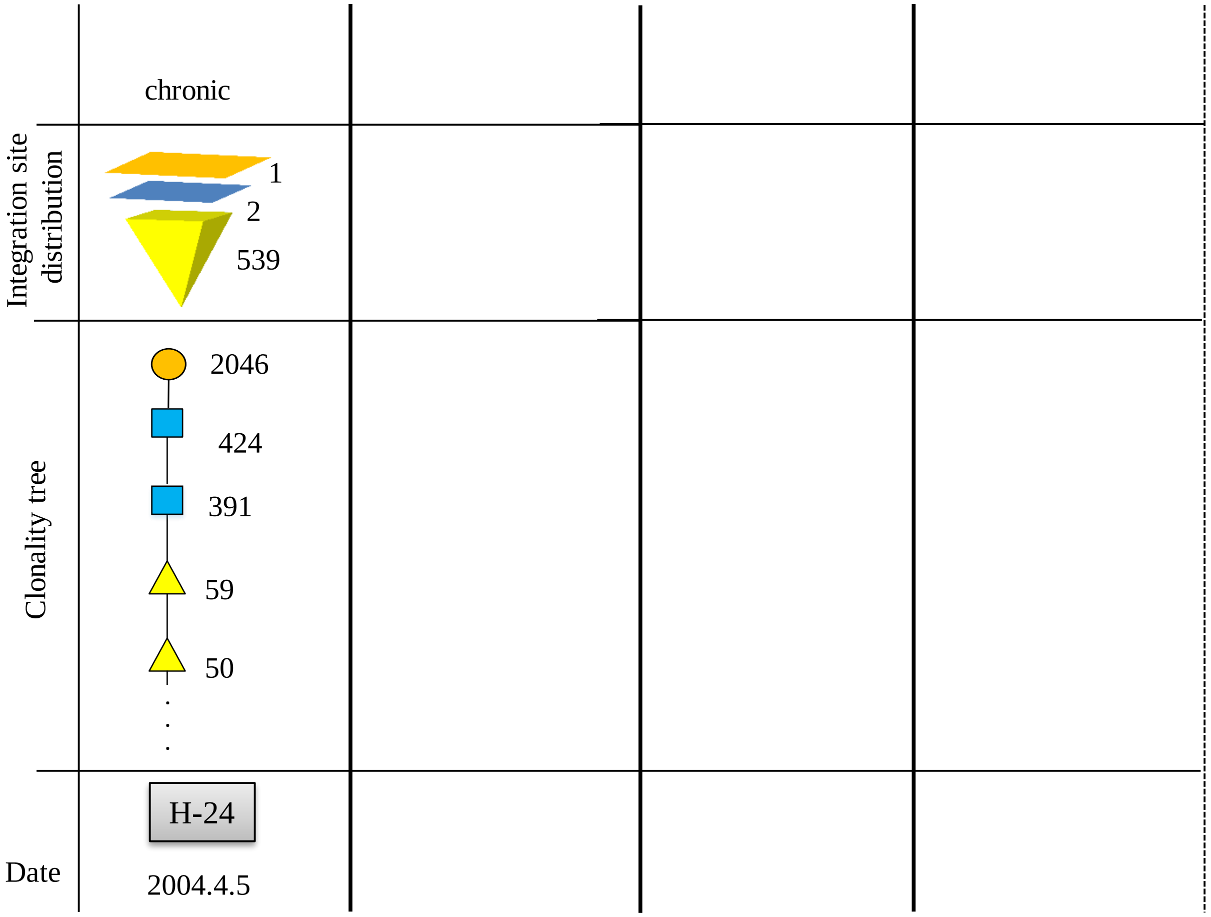
chronic
Integration site distribution
1
2
539
Clonality tree
2046
424
391
59
50
H-24
Date
2004.4.5
