# Supplementary material for: Inferring clonal structure in HTLV-1-infected individuals: towards bridging the gap between analysis and visualization
Source: Hum Genomics. 2017 Jul 11;11:15. doi: 10.1186/s40246-017-0112-8 (PMC5505134; doi:10.1186/s40246-017-0112-8)
Supplement: Supplementary file 3 — Disease status and clonal analysis over time. (PDF 234 kb) [file 40246_2017_112_MOESM3_ESM.pdf]

Table S2: Disease status and clonal analysis over time

Disease status	Size of largest clone	Clonality Pattern	Analyzed samples
AC→AC	VS, S	Poly	H-3→H-1, H-9→H-7, H-2→H-4, H-8→H-5
AC→SM	B, VB	Oligo, Mono	H-20→H-16→H-18→H-15,H-28→H-32→H41
AC→Chronic→Acute	B,VB	Mono	H-19→H-25→H-53→H-57
AC→SM→Chronic	B, VB	Mono	H-34→H-31→H-52→H-42
SM→Chronic	B, VB	Oligo, Mono	H-22→H-36,H-13→H-38,H-40→H-33,H-51→H-45,H-14→H-23
SM→SM	B, VB	Mono	H-6→H-27
SM→Acute	VB	Mono	H49→H-50
Chronic→Chronic	VB	Mono, Oligo	H-54→H-56, H46→H43, H47→H-44
Chronic→Acute	VB	Mono	H-60→H-59
Acute under treatment	VB	Mono	H-30→H-39→H-26→H-55

Asymptomatic carriers (ACs) with no progression had only VS and S clones and represented a polyclonal pattern. Acute samples had only VB clones and represented a monoclonal pattern. Smoldering (SM) and chronic patients (indolent types of ATL) had B and/or VB clones. Detailed information for each sample is provided in Additional file 1: Table S1.
